# TIP peptide inhalation in experimental acute lung injury: effect of repetitive dosage and different synthetic variants

**DOI:** 10.1186/1471-2253-14-42

**Published:** 2014-05-26

**Authors:** Erik K Hartmann, Rainer Thomas, Tanghua Liu, Joanna Stefaniak, Alexander Ziebart, Bastian Duenges, Daniel Eckle, Klaus Markstaller, Matthias David

**Affiliations:** 1 Department of Anaesthesiology, Medical Centre of the Johannes Gutenberg-University Mainz, Langenbeckstraße 1, 55131 Mainz, Germany; 2 Department of Anaesthesiology, General Critical Care Medicine and Pain Therapy, Medical University Vienna, Währinger Gürtel 18-20, 1090 Vienna, Austria

**Keywords:** TIP peptide, Lectin-like domain, Pulmonary oedema, Alveolar fluid clearance, ARDS, Porcine model

## Abstract

**Background:**

Inhalation of TIP peptides that mimic the lectin-like domain of TNF-α is a novel approach to attenuate pulmonary oedema on the threshold to clinical application. A placebo-controlled porcine model of acute respiratory distress syndrome (ARDS) demonstrated a reduced thermodilution-derived extravascular lung water index (EVLWI) and improved gas exchange through TIP peptide inhalation within three hours. Based on these findings, the present study compares a single versus a repetitive inhalation of a TIP peptide (TIP-A) and two alternate peptide versions (TIP-A, TIP-B).

**Methods:**

Following animal care committee approval ARDS was induced by bronchoalveolar lavage followed by injurious ventilation in 21 anaesthetized pigs. A randomised-blinded three-group setting compared the single-dosed peptide variants TIP-A and TIP-B as well as single versus repetitive inhalation of TIP-A (n = 7 per group). Over two three-hour intervals parameters of gas exchange, transpulmonary thermodilution, calculated alveolar fluid clearance, and ventilation/perfusion-distribution were assessed. Post-mortem measurements included pulmonary wet/dry ratio and haemorrhage/congestion scoring.

**Results:**

The repetitive TIP-A inhalation led to a significantly lower wet/dry ratio than a single dose and a small but significantly lower EVLWI. However, EVLWI changes over time and the derived alveolar fluid clearance did not differ significantly. The comparison of TIP-A and B showed no relevant differences. Gas exchange and ventilation/perfusion-distribution significantly improved in all groups without intergroup differences. No differences were found in haemorrhage/congestion scoring.

**Conclusions:**

In comparison to a single application the repetitive inhalation of a TIP peptide in three-hour intervals may lead to a small additional reduction the lung water content. Two alternate TIP peptide versions showed interchangeable characteristics.

## Background

Within the course of Acute Respiratory Distress Syndrome (ARDS) neutrophil accumulation and a developing, non-cardiogenic alveolar oedema represent early pathophysiologic alterations, which are followed by an impaired resolution of the accumulated fluid [1]. A widespread array of aetiologies that induce endothelial or alveolar epithelial damage can cause ARDS. This results in increased microvascular permeability and disruption of the alveolar-capillary unit with a consecutively impaired ion and fluid transfer across the injured alveolar epithelium [1-3]. Moreover, there is growing evidence that the clearance of the oedema fluid by the lung itself is an important factor in overcoming ARDS [4-7]. Tumor-necrosis-factor-α (TNF-α) plays an important but dichotomal role in early oedema formation: on the one hand TNF-α promotes receptor-mediated inflammatory response in injured lungs [8,9]. The receptor-independent lectin-like domain of TNF-α, on the other hand, is responsible for beneficial effects: pulmonary application of TIP peptides mimicking this lectin-like domain increases oedema reabsorption via sodium transfer across the alveolar epithelium and also reduces microvascular permeability, which prevents further oedema formation [10-13]. The lectin-like domain therefore represents a novel approach in pharmacologic treatment of ARDS [3].

An application of a TNF-α derived TIP peptide (TIP-A) led to a sustained reduction of the lung water content in previous studies [10,12,14], though a repetitive application has not yet been examined in vivo. An alternate, synthetic version of the TIP peptide (TIP-B) was slightly more effective in in vitro tests [15,16], but it is unknown, if this effect is transferable to an in vivo setting. Following a study that demonstrated oedema-attenuating properties of TIP peptide inhalation in a porcine model [14] we examined two hypotheses: (1) Single doses of two synthetic peptide variants (SD-TIP-A, SD-TIP-B) show comparable effects on lung water content and the pulmonary function. (2) Repetitive application of TIP-A (RD-TIP-A) further reduces lung water content and improves gas exchange in comparison to a single dose.

## Methods

Following approval of the state and institutional animal care committee (Landesuntersuchungsamt Rheinland-Pfalz, Koblenz, Germany; 23 177–07/G 09-1-045) 21 healthy juvenile pigs (24–27 kg) were examined in a randomised, investigator-blinded setting.

### Anaesthesia and instrumentation

The animals were sedated by an intramuscular injection of midazolam and ketamine. General anaesthesia was induced and maintained by intravenous administration of propofol (Fresenius Kabi, Bad Homburg, Germany; 4 mg kg^-1^ followed by 8–12 mg kg^-1^ h^-1^) and fentanyl (Janssen-Cilag, Neuss, Germany; 4 μg kg^-1^ followed by 0.1-0.2 mg h^-1^). Endotracheal intubation was performed and pressure-controlled ventilation was initiated: tidal volume 10 ml kg^-1^, positive end-expiratory pressure (PEEP) 5 cmH_2_O, fraction of inspired oxygen (FiO_2_) 0.3-0.4 and respiratory rate targeted to achieve normocapnia. Extended haemodynamic monitoring was established surgically: a pulmonary artery catheter, an arterial line for blood pressure monitoring, a central venous line and a pulse contour cardiac output catheter (PiCCO, Pulsion Medical, Munich, Germany). Following the instrumentation a fluid optimisation routine consisting of 50 ml of hydroxyethyl starch (130/0.4, Volulyte, Fresenius Kabi, Bad Homburg, Germany) was conducted. All animals received a background infusion of balanced electrolyte solution (Sterofundin, B.Braun, Melsungen, Germany, 5 ml kg^-1^ h^-1^).

### Lung injury model

Lung injury was induced by bronchoalveolar lavage followed by a constant, injurious ventilation mode: pressure-controlled ventilation, tidal volume 10 ml kg^-1^, PEEP 0 cmH_2_O, FiO_2_ 1.0, respiratory rate 25–35 min^-1^ targeted to a carbon dioxide level < 8 kPa. The bronchoalveolar lavage was performed as previously described [14]. The amount of instilled and recovered fluid was recorded to determine the fluid intake by lavage. Lavage procedures were repeated until a ratio of arterial partial pressure of oxygen (PaO_2_) and FiO_2_ ≤ 200 mmHg was achieved and maintained for 45 minutes. Afterwards baseline measurements were taken.

### Protocol and drug administration

The TNF-α derived TIP peptides A and B (AP301 and AP318, APEPTICO, Vienna, Austria) were delivered by the manufacturer as lyophilisate at – 20° Celsius. Every single dosage (1 mg kg^-1^) was dissolved at the day of application in 5 ml water for injection, which resulted in a transparent, non-discriminable fluid for inhalative application. We used a commercially available, vibrating-plate nebulizer (Aeroneb ProX, Aerogen, Galway, Ireland) that was previously bench tested for TIP peptide inhalation by APEPTICO. Randomisation and preparation of the peptides were conducted by a non-participant for blinded administration:

(1) SD-TIP-A group (n = 7; 1 mg kg^-1^ AP301 at 0 h, vehicle at 3 h).

(2) RD-TIP-A group (n = 7; 1 mg kg^-1^ AP301 at 0 h and 3 h).

(3) SD-TIP-B group (n = 7; 1 mg kg^-1^ AP318 at 0 h, vehicle at 3 h).

### Measured parameters

The animals were monitored for six hours after the first TIP nebulisation. Spirometry, gas exchange and haemodynamics were continuously monitored. The ventilation/perfusion-distribution (V/Q) was determined as percentage of cardiac output by micropore membrane inlet mass spectrometry - multiple inert gas elimination technique (MMIMS-MIGET, Oscillogy LLC, Folsom, USA) according to a previously reported routine [17]. Development of pulmonary oedema was assessed by the transpulmonary thermodilution-derived extravascular lung water index (EVLWI [ml kg^-1^]; PiCCO, Pulsion Medical, Munich, Germany) and post-mortem wet/dry ratio (W/D). The alveolar fluid clearance (AFC [%]) was calculated as reported by Garcia-Delgado and co-workers [18] with a modification that uses the amount of non-recovered lavage fluid as reference: (EVLW_Baseline_ [ml] – EVLW_6h_ [ml]) × 100/non-recovered lavage fluid [ml]. At the end of the experiment the animals were killed in general anaesthesia by central venous injection of propofol (200 mg) and potassium chloride (40 mval). The lung was removed en-bloc after thoracotomy and assessed by a lung injury score based on haemorrhage und congestion of the lung surface [19]. The complete lung surface was divided in four ventral and four dorsal segments (each upper/lower right, upper/lower left) and each segment was scored individually: 2 points for haemorrhage and congestion > 50% of the surface, 1 point for < 50%, 0 points for no or minimal, which results in a maximum score value of 16 points. Afterwards the exsanguinated left lung was weighted, sliced and dried for determination of the W/D ratio.

### Statistical analysis

Data are expressed as median and interquartile range (IQR). According to the two predefined hypotheses comparisons were only drawn SD- versus RD-TIP-A and SD-TIP-A versus SD-TIP-B. Repetitively measured parameters (ARDS baseline, three and six hours) were analysed by Friedman Analysis of Variance and SNK-Test for multiple comparisons. For non-recurring parameters the Mann–Whitney-U-Test was used. P-values < 0.05 were regarded as significant. Additionally, we conducted a post-hoc comparison of the EVLWI values and their changes over time to a previously published vehicle group [14] from the identical ARDS model. The statistical software SigmaPlot 11.0 (Systat Software, Erkrath, Germany) was used.

## Results

### Baseline parameters

All three groups received a comparable number of lavage procedures (SD-TIP-A: 3 (1); RD-TIP-A: 3 (2); SD-TIP-B: 3 (1)). The amounts of fluid that were not recovered from the lungs during lavage were 360 (155) ml (SD-TIP-A), 320 (105) ml (RD-TIP-A), and 350 (115) ml (SD-TIP-B). The small differences were not statistically significant. The ARDS baseline data are summarized in Table 1.

**Table 1:** **Additional parameters and MMIMS-MIGET-derived** V/Q **over six hours**

	**SD-TIP-A**			**RD-TIP-A**			**SD-TIP-B**		
**Parameter**	**Baseline**	**3 h**	**6 h**	**Baseline**	**3 h**	**6 h**	**Baseline**	**3 h**	**6 h**
AaDO_2_ [kPa]	64.7 (4.3)	50.4 (10.3)	49.6 (8.8)	66.4 (6.3)	50.9 (20.3)	45.1 (17.5)	71.3 (11.5)	52.4 (14.0)	42.5 (14.3)
PaCO_2_ [kPa]	6.7 (1.0)	5.1 (2.3)	5.5 (0.7)	7.5 (2.5)	6.5 (0.5)	6.0 (0.8)	7.2 (1.0)	6.1 (0.7)	5.7 (0.5)
Shunt [%]	35 (7)	20 (2)	25 (7)	28 (8)	20 (3)	19 (9)	33 (15)	19 (7)	20 (6)
Low V/Q [%]	0 (2)	6 (15)	0 (3)	3 (6)	0.1 (7.5)	0 (0)	0 (0)	5 (15)	0 (0)
Normal V/Q [%]	62 (8)	70 (11)	72 (5)	67 (6)	74 (13)	79 (8)	65 (11)	72 (17)	79 (6)
High V/Q [%]	0.7 (0.2)	0.6 (0.3)	0.6 (0.9)	0.7 (0.8)	0.7 (0.4)	1.0 (0.8)	0.6 (1.1)	0.7 (0.8)	0.5 (1.5)
V_t_ [ml kg^-1^]	9.8 (0.2)	10.1 (0.5)	9.9 (0.6)	10.2 (0.6)	10.3 (0.7)	10.1 (0.5)	9.7 (0.2)	9.9 (0.6)	9.8 (0.8)
P_peak_ [cmH_2_O]	20 (4)	20 (4)	19 (5)	20 (6)	20 (11)	19 (8)	19 (4)	23 (8)	19 (7)
PEEP [cmH_2_O]	0.5 (0.1)	0.6 (0.7)	0.6 (0.2)	0.5 (0.2)	0.6 (0.6)	0.6 (0.5)	0.6 (0.2)	0.6 (0.3)	0.6 (0.3)
RR [min^-1^]	30 (4)	28 (5)	30 (5)	30 (4)	30 (4)	30 (3)	30 (6)	30 (3)	29 (5)
C_dyn_ [ml mbar^-1^]	13 (3)	13 (3)	13 (4)	13(3)	13 (4)	13 (4)	14 (3)	13 (4)	11 (5)
R_aw_ [mbar s l^-1^]	8 (1)	8 (2)	8 (1)	8 (2)	9 (3)	9 (3)	7 (1)	7 (2)	8 (2)
MAP [mmHg]	100 (6)	93 (15)	87 (19)	102 (25)	92 (14)	91 (11)	89 (11)	85 (18)	80 (15)
MPAP [mmHg]	30 (5)	27 (4)	26 (6)	35 (6)	31 (5)	30 (7)	31 (7)	29 (12)	26 (14)
CO [l min^-1^]	3.8 (1.0)	3.5 (1.2)	3.3 (0.9)	3.8 (0.9)	3.6 (0.7)	3.5 (0.5)	3.7 (1.2)	3.6 (0.6)	3.3 (0.9)
PVR [dyn s cm^-5^]	423 (127)	384 (152)	432 (368)	362 (122)	395 (137)	436 (120)	392 (199)	333 (252)	411 (355)
HR [min^-1^]	100 (25)	97 (18)	96 (23)	109 (36)	92 (39)	96 (36)	114 (27)	100 (33)	100 (23)
PCWP [mmHg]	13 (5)	12 (6)	13 (7)	14 (5)	13 (4)	13 (6)	12 (2)	12 (5)	12 (5)
CVP [mmHg]	11 (1)	10 (2)	10 (2)	12 (2)	11 (4)	10 (4)	10 (1)	10 (1)	10 (1)

Data are expressed as median (IQR). No relevant intergroup differences were found. *AaDO*_*2*_: Alveolar-arterial oxygen gradient; *PaCO*_*2*_: Arterial partial pressure of oxygen; Shunt and V/Q measured by *MMIMS-MIGET*; *V*_*t*_: Tidal volume; *P*_*peak*_: Peak inspiratory pressure; *PEEP*: Positive end-expiratory pressure; *RR*: Respiratory rate; *FiO*_*2*_: Fraction of inspired oxygen; *C*_*dyn*_: Dynamic compliance; *R*_*aw*_: Airway resistance; *MAP*: Mean arterial pressure; *MPAP*: Mean pulmonary arterial pressure; *CO*: Cardiac output; *PVR*: Pulmonary vascular resistance; *HR*: Heart rate; *PCWP*: Pulmonary capillary wedge pressure; *CVP*: Central venous pressure.

### Effect upon lung water content

**Figure 1:**
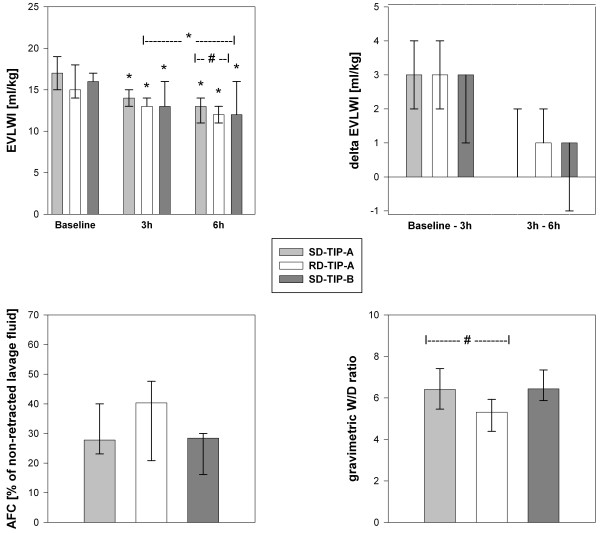
**Assessment of the lung water content.** The figure shows the thermodilution-derived extravascular lung water index (EVLWI), the EVLWI changes over time (delta EVLWI), the calculated alveolar fluid clearance (AFC) over six hours, and the post-mortem gravimetric wet/dry (W/D) ratio of the lung. Bars represent median (IQR). * indicate P < 0.05 versus the baseline or 3 h-value, # indicates P < 0.05 in the intergroup comparison. According to the study’s hypotheses no comparisons between RD-TIP-A and SD-TIP-B were drawn.

Figure 1 shows the assessment of the lung water content. The W/D ratio yields a significantly lower value of 5.3 (1.2) in the RD-TIP-A-group, while the median values are 6.4 (1.0) in the SD-TIP-A-group and 6.4 (1.1) in the SD-TIP-B-group. In all three groups the EVLWI significantly decreases over six hours. The main reduction occurs after the first TIP inhalation in all groups. Only the RD-TIP-A-group shows a small further reduction within hours 3–6. At six hours a significant difference was found between SD- and RD-TIP-A. But the corresponding AFC and overall EVLWI changes over time ((∆ EVLWI) [ml kg^-1^]; RD-TIP-A: 4 (2); SD-TIP-A: 4 (1); SD-TIP-B: 3 (1)) did not differ between the groups. The post-hoc comparison to a previous control group (EVLWI_Baseline_ 17 (5), EVLWI_5h_ 16 (2), ∆ EVLWI 1 (2); from [14]) demonstrates a significantly lower absolute and higher ∆ EVLWI in all three TIP groups over five hours (SD-TIP-A: EVLWI_5h_ 14 (2), P = 0.040; RD-TIP-A EVLWI_5h_ 12 (3), P = 0.001; SD-TIP-B: EVLWI_5h_ 14 (3), P = 0.019) without differences at baseline.

### Influence on oxygenation and ventilation-/perfusion-distribution

**Figure 2:**
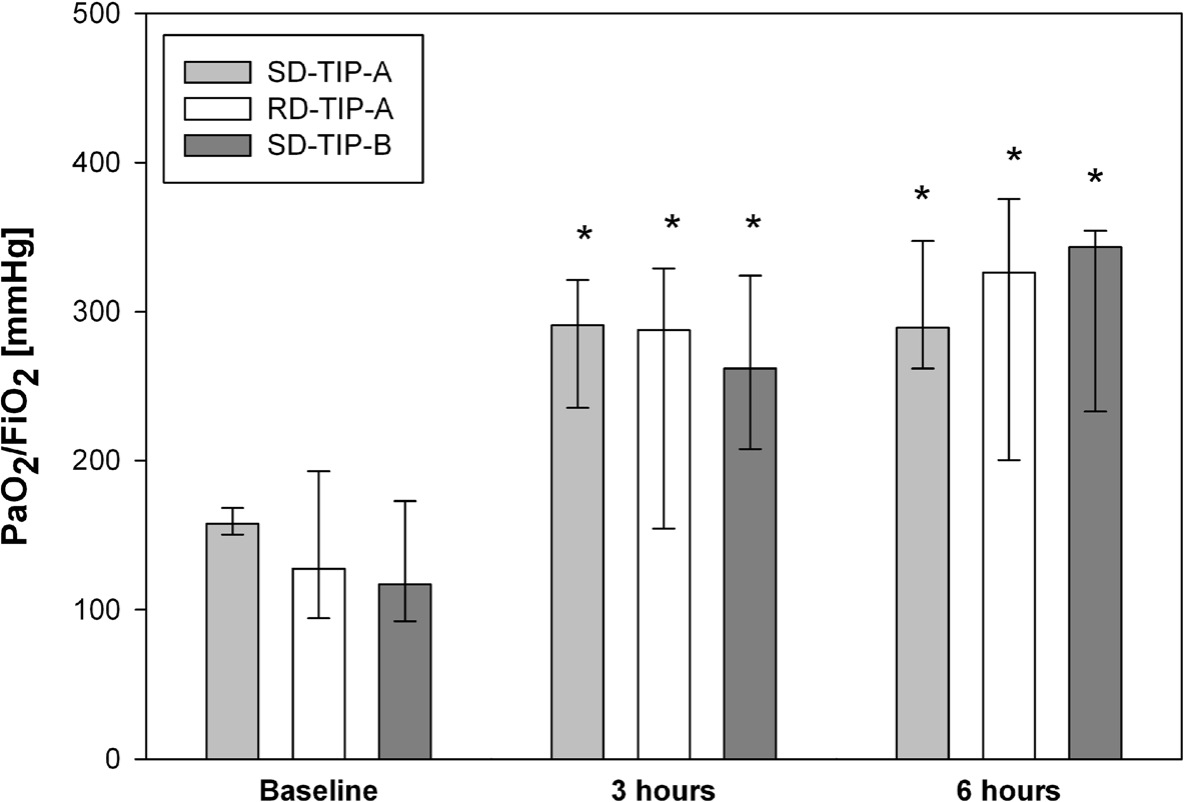
**Time course of the PaO**_**2**_**/FiO**_**2 **_**quotient in all three groups.** Bars represent median (IQR). * indicate P < 0.05 vs. baseline value. The three groups do not differ significantly at any time point (P > 0.05).

**Figure 3:**
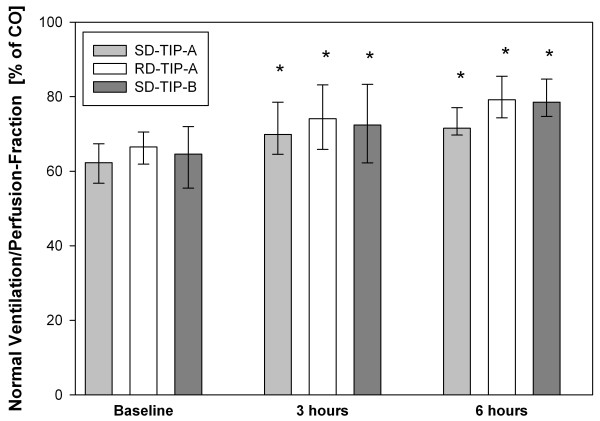
**Normal ventilation/perfusion-distribution by MMIMS-MIGET after six hours.** Bars represent median (IQR). * indicate P < 0.05 vs. baseline value. No intergroup differences occurr (P > 0.05).

The PaO_2_/FiO_2_ ratio considerably increased over six hours in all three groups (Figure 2), which mainly occurred within the hours 1–3. The three groups did not differ at any time point. The MMIMS-MIGET derived V/Q is summarized in Table 1 and Figure 3. In all groups the normal V/Q fraction recovered over six hours (each P < 0.05), which was caused by a decrease of the combined shunt and low V/Q fraction.

### Haemorrhage/congestion scoring and additional parameters

**Figure 4:**
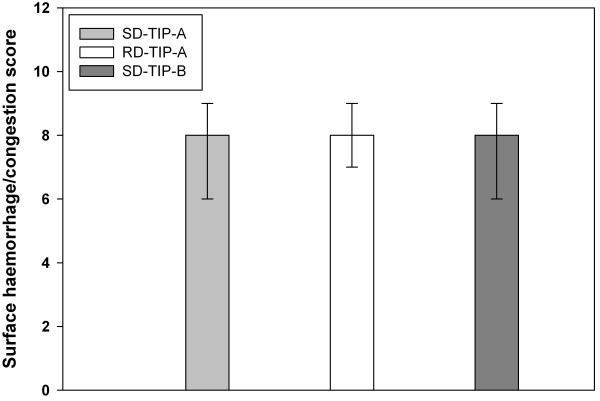
**Results of the post-mortem haemorrhage/congestion scoring.** Bars represent median (IQR). The data show no intergroup differences (P > 0.05).

The score values are 8 (3) in the SD-TIP-A-group, 8 (2) in the RD-TIP-A-group, and 8 (3) in the SD-TIP-B-group (Figure 4). Table 1 summarizes the monitored parameters during the observation period. Neither haemodynamic instability occurred nor was vasopressor support required. Respiratory parameters and mechanics showed no relevant differences.

## Discussion

The present experimental study features two main findings: (1) an only small further attenuation of pulmonary oedema due to repeated TIP peptide inhalation in comparison to a single dose in a pre-validated porcine model and (2) no differences in comparison of two single-dosed TIP peptide versions (TIP-A and TIP–B) with respect to lung water content, gas exchange and V/Q distribution.

### Model characteristics

The early course of ARDS is characterized by pulmonary fluid accumulation, reduction of lung compliance and impaired gas exchange. Several experimental models can be used to simulate distinct pathophysiologic components of ARDS, though none mimics the entire pattern of human ARDS. Repetitive saline lung lavage depletes surfactant, but causes only mild structural damage of the alveolar epithelium and low-grade injury of the alveolar-capillary unit [20-22]. Repetitive lavage alone hardly mimics the full pattern of ARDS. The combination with an injurious ventilation mode, however, should induce ongoing epithelial barrier damage and is propagated to be more similar to human ARDS [20,23]. However, standardized protocols to achieve this are lacking. We chose a mild combination of lavage-induced lung injury and injurious ventilation (tidal volume 10 ml kg^-1^, PEEP 0 cmH_2_O): this approach warrants a relatively constant state of gas exchange impairment and oedema without further therapy [14]. The main rationale is that the apical side of the alveolar epithelium represents the TIP peptide’s primary target [11]. The peptide’s impact upon longer established or severe ARDS may consequently be limited through a sustained epithelial necrosis and its deposition only in ventilated areas [24].

The lavage procedure also leads to alveolar flooding and pulmonary fluid accumulation (in the present study about 300 ml). Vadasz and co-workers induced lung injury and alveolar flooding in isolated ventilated and perfused rabbit lungs by an elevation of pulmonary venous pressure or endo-/exotoxin administration [12]. They demonstrated that single doses of a TIP peptide reduced vascular permeability and increased the absorption of intra-alveolar fluid. Similar results were found in an ex vivo flooded rat lung model [10].

### Comparison of TIP-A and B

The TIP peptide AP301 stimulates the sodium influx in human A549 cells and type II alveolar cells of several animal species, which creates an osmotic gradient from the alveoli to the interstitium. In porcine cell layers an approximately 16-fold increase of sodium incurrent occurred within fifteen minutes after TIP peptide application [25]. Several synthetic peptides mimicking the lectin-like domain of TNF-α activate epithelial sodium channels, which were identified as the key structures targeted by TIP peptides [15]. TIP-B was slightly more effective in in vitro experiments [15,16]. But we detected no relevant differences regarding gas exchange and lung water content between the two TIP peptide versions in vivo. Prior to this the significant advantage of TIP-A over placebo was demonstrated in the identical ARDS model [14]: a post-hoc comparison to this study’s control group shows a significant EVLWI reduction for SD-TIP-A and SD-TIP-B. Beneficial effects on gas exchange in vivo were also shown in a rat lung-transplantation model [11]. Our first hypothesis was therefore approved.

### Repetitive dosage

The time courses following a single TIP dose were in line with the prior report. In contrast, the repetitive application of TIP-A led to a considerably lower W/D ratio than the single dose and an only small but further reduction of the EVLWI, whereas the ∆ EVLWI and derived AFC did not differ significantly between SD- and RD-TIP-A. Our overall AFC was lower than reported in healthy pigs (around 50% over four hours without PEEP [18]), which fits to the concept of impaired AFC in ARDS. Though, the used formula does not consider, if an injurious ventilation mode leads to ongoing oedema formation. Another porcine ARDS model, however, demonstrated considerably lower AFC rates [26]. The present data imply that the impact of the TIP peptide on the lung water content may be short-termed but traceable even under sub-standard ventilation. However, pharmacologic reduction of lung water content alone may not improve outcome or mortality [27]. The recovery of lung function in terms of oxygenation and V/Q was not intensified by repetitive inhalation. Therefore our second hypothesis was only partially confirmed: the observed, additional changes of the lung water content are quite small and may not be sufficient to cause amelioration of gas exchange. The majority of the effect is definitely attributed to the first TIP inhalation in all groups (Figure 1). In this context, it is worth to note that the present study was not designed to analyse synergistic effects with the current standard of lung-protective ventilation. To some extent the present model and ventilation mode will not restore healthy conditions without any intervention [14], which is also be reflected by the considerable presence of haemorrhage and congestion in all groups.

### Implications of oedema reabsorption

The V/Q data suggest that the impaired oxygenation is caused mainly by intrapulmonary shunt and low V/Q units from collapsed or flooded alveoli. Similar results were reported in two other porcine lavage models [17,28]. Due to the ventilation mode without PEEP a sustained recruitment of non-ventilated lung areas over time is unlikely, which is consistent with the persisting shunt fraction and the minimal presence of high V/Q units indicating hyperinflation. The present study describes two different effects of the TIP peptides: optimization of the V/Q mismatch and reduced lung water content. Recent studies characterised the correlations between EVLWI, the amount of non-aerated lung tissue, and V/Q distribution in pigs [29,30]. Based on the present data we can hardly differentiate, if oedema reduction and improved V/Q matching are associated or occur merely simultaneous. Previous studies did not report on systemic or haemodynamic effects of the TIP peptide [11,31]. Hence, a relevant impact of the peptide itself on pulmonary perfusion is unlikely. The physiological hypoxic pulmonary vasoconstriction, however, is unaffected after lung lavage [32].

Recovery from ARDS requires reabsorption of the pulmonary oedema and enabling of the epithelial structures to rest and recover [33]. Distribution of pulmonary oedema in ARDS is not homogeneous. The injured lung is therefore widely inhomogeneous in its response to mechanical ventilation or recruitment even during lung protective ventilation [34,35]. This can result in increased cyclic stretch and strain of the lung tissue, which is associated with vascular permeability and inflammation. Patients suffering from a high-grade AFC impairment in early ARDS have significantly higher mortality rates [4]. The present model implies a sustained effect of the TIP peptide on the initial alveolar flooding, which, in the absence of recruitment or PEEP [18], is consistent with a resolution of oedema fluid. Hence, an early enhancement of the alveolar fluid clearance may not only reduce the lung water content but also lead to more homogenous lung tissue. This could positively affect distribution of mechanical ventilation and stress within the lung tissue.

### Limitations

TIP peptide inhalation currently lacks an established dose-effect correlation in vivo. Therefore, we extrapolated the applied dosages of 1 mg kg^-1^ from previous small animal and ex vivo models (approximately 0.5 mg kg^-1^, [10,11]). Subsequently, this dosage was effective in a previous placebo-controlled porcine study [14]. Accordingly we passed on an additional placebo group that would merely repeat this previous work without an impact on this study’s two hypotheses. However, we performed a post-hoc comparison of the present study’s EVLWI data to this previous control group. The W/D ratio was calculated in a previously reported manner without correction for pulmonary blood content in exsanguinated pigs [22], which should though primarily reflect alveolar oedema [36]. The majority of the TIP peptide’s effect occurred within three hours after application. A persisting influence of the TIP peptide upon intrapulmonary inflammation was assumed in a recent study [11]. Accumulation of neutrophil granulocytes and generation of reactive oxygen species were significantly attenuated by a TIP peptide [11,13]. Potential anti-inflammatory effects were not analysed, but may be equalized by the ongoing mechanical stress resulting from an injurious ventilation mode. Altogether, the present study was designed to characterise acute effects in a relatively mild ARDS and not to provide long-term outcome data, which rather is the domain of early clinical studies.

## Conclusions

In a model of lung lavage followed by constant injurious ventilation the repetitive inhalation of a TNF-α derived TIP peptide may lead to a small additional decrease of lung water content. Though, this repetitive dosage did not improve gas exchange or attenuate pulmonary haemorrhage and congestion in comparison to a single dose. Inhaled TIP peptides induce a clearance of pulmonary oedema and improve the ventilation/perfusion-distribution. Two alternate versions of the TIP peptide showed interchangeable characteristics in vivo.

## Abbreviations

AFC: Alveolar fluid clearance; ARDS: Acute respiratory distress syndrome; EVLWI: Extravascular lung water index; FiO_2_: Fraction of inspired oxygen; IQR: Interquartile range; MMIMS-MIGET: Micropore membrane inlet mass spectrometry – multiple inert gas elimination technique; PaO_2_: Arterial partial pressure of oxygen; PEEP: Positive end-expiratory pressure; RD: Repetitive dosage; SD: Single dosage; TNF-α: Tumor-necrosis-factor-α; V/Q: Ventilation/perfusion distribution; W/D: Pulmonary wet/dry ratio.

## Competing interests

All experiments were performed at the Department of Anaesthesiology, Medical Centre of the Johannes Gutenberg-University, Mainz, Germany. The study was funded in part by APEPTICO, Vienna, Austria, which developed the peptides AP301 and AP318. APEPTICO had no influence on the performance of the experiments, data analysis and interpretation or manuscript drafting.

## Authors’ contributions

EKH coordinated and supervised the experiments. EKH, RT, TL, JS and AZ conducted the experiments. EKH, RT, BD and DE performed the data analysis. EKH drafted the manuscript. KM and MD participated in the study design, supervision of laboratory, data analysis and revision of the manuscript. All authors edited and approved the final manuscript.

## Pre-publication history

The pre-publication history for this paper can be accessed here:

http://www.biomedcentral.com/1471-2253/14/42/prepub
